# Immune Evasion Through Human Leukocyte Antigen Implications and Its Impact on Targeted Therapy

**DOI:** 10.7759/cureus.52737

**Published:** 2024-01-22

**Authors:** Mihaela Andreescu, Bogdan Andreescu

**Affiliations:** 1 Faculty of Medicine, Titu Maiorescu University, Bucharest, ROU; 2 Hematology, Colentina Clinical Hospital, Bucharest, ROU; 3 Plastic Surgery, Colentina Clinical Hospital, Bucharest, ROU

**Keywords:** hla, natural killer cells, kir, targeted therapies, immune evasion

## Abstract

The malfunctioning of human leukocyte antigen (HLA) class I antigens has a substantial negative impact on the effectiveness of leukemia treatment, particularly in the development of immunotherapies that rely on T-cell activation. HLA-G, a molecule that suppresses the immune response, plays a role in repressing the activation and proliferation of T cells, natural killer cells, and antigen-presenting cells. The expression of HLA-G is associated with various pathological conditions. Tumor cells exploit the immune evasion capabilities of HLA, allowing them to evade detection and elimination by the immune system. Understanding and modifying the HLA molecules is crucial for the advancement of innovative immunotherapies targeting chronic lymphocytic leukemia. Numerous mechanisms have been investigated to elucidate how HLA facilitates tumor evasion in patients with chronic lymphocytic leukemia and other malignancies. These mechanisms include inhibiting immune cell cytolysis, altering cytokine production levels, promoting immune cell programmed cell death, and impairing chemotaxis. This review provides a comprehensive overview of immune evasion mediated by HLA and its implications for targeted therapy.

## Introduction and background

The HLA class I antigens are essential in interacting virus-affected and cancerous cells with effector cells. HLA class I molecules act as mediators by presenting targeted cancer cells to cytotoxic T lymphocytes. This enables the immune system to identify and eliminate tumor cells by recognizing tumor-associated antigens on the surface of cancer cells. However, some tumors can evade detection and persist by developing resistance mechanisms, such as modifying HLA expression. This alteration hinders the presentation of tumor-related antigens to immune cells, enabling the tumors to escape immune surveillance [1]. The activation or deactivation of the immunoglobulin receptors present on the surface of natural killer (NK) cells through their interaction with HLA molecules triggers the activation of these cells [2]. The HLA molecule with functional and antigenic defects is present in chronic lymphoma patients' cell lines and also in tumors extracted from lesions. The prevalence of these defects varies among different tumor types. Currently, the characterization of HLA class I antigen expression in tumor cells and the mechanism of the defect in HLA class I antigen is a prime factor for immune evasion research, as HLA class I defects have a negative influence on immunotherapy. The study of the depiction of HLA class I antigen expression in hematological cancers, for instance, B-cell lymphomas [3], chronic lymphoblastic leukemia (CLL) [4], and other leukemias such as acute lymphoblastic leukemia (ALL) and acute myeloid leukemia (AML), is limited [5]. This article offers a comprehensive review of the impact of immunological evasion through HLA implications and the impact this has on the response to targeted therapy.

## Review

Mechanism of immune evasion

The immune system can generate effective T cells for the lysis of tumors. However recent research has shown that cancerous cells can evade the immune system by several mechanisms. These evasion strategies involve the activation of antiapoptotic factors and the inhibition of pro-apoptotic factors, allowing tumor cells to escape predation by CD8+ cells and cytotoxic lymphocytes. Furthermore, alterations in HLA molecules and other co-inhibitory molecules contribute to immune evasion. For example, programmed death ligands play a role in shielding cells from immune scrutiny by evading both NK-cell and cytotoxic lymphocyte-mediated responses [6].

Suppression of HLA class I antigen and HLA-mediated escape mechanisms

HLA class I antigen loss and downregulation have been demonstrated in several tumors. There are several mechanisms responsible for HLA-mediated immune evasion, including deficiency of beta 2 microglobulin or lack of antigen processing expression associated transporter [7], loss of heterozygosity (LOH), removal of genes in chromosomes, or mitotic makeup in chromosome 6 [8], downregulation of transcription factors, single nucleotide mutation [9], and partial chromosomal depletion, or somatic recombination [10]. Demanent et al., in their study, examined the expression of HLA class I alleles in leukemic cells using a panel of human monoclonal antibodies for HLA class I allospecificity identification. The comparative analysis of 116 HLA class I alleles in thirty-two samples of leukemic cells and their corresponding normal cells revealed a significant downregulation of HLA-A and HLA-B alleles in 35% and 38% of cases, respectively. Notably, the HLA-Bw6 determinant, which is located within the HLA class I region, exhibited more pronounced downregulation. This finding is particularly intriguing due to the interaction between the HLA and the Bw4 and Bw6 alleles NK cell receptors. However, unlike HLA-C allospecifics, the presence of HLA-B alleles does not inhibit NK cell toxicity. The HLA-B alleles are shielded from NK cell attack by the presence of the HLA Bw6 epitope. Consequently, it can be inferred that the downregulation of HLA-Bw6 enables leukemic cells to evade immune recognition and subsequent cytotoxic T-cell attacks. This suggests that immunotherapeutic strategies should focus on the use of HLA-Bw6 alloantigens as regulating elements to prevent the escape of leukemic cells from cytotoxic T cells, as the forfeiture of HLA-Bw4 allele would make them inclined to NK-cell mediated lysis [11].

Immune evasion through HLA LOH

Masuda et al. carried out a study on cell lines that underwent LOH to investigate the LOH haplotype [12], and many other researchers studied tissue from solid tumors [13,14]. They concluded that haplotype loss of HLA is the culprit for immune evasion in leukemic cells, as it happens in solid tumors. However, results have suggested that HLA gene haplotype loss occurs more seldom in leukemic cells than in solid tumors. Specifically, studies have shown that leukemic cell lines, particularly those associated with lymphocytic leukemia, exhibit a loss of expression of HLA class I molecules [15,16]. In contrast, solid tumors tend to exhibit a lower incidence of LOH or suppression of HLA expression compared to leukemic blasts. For instance, skin cancer, large intestine carcinoma, renal cell cancer, and laryngeal cancer display LOH or HLA suppression in approximately 20% to 70% of cases [17,18]. In breast cancer, HLA downregulation accounts for 38% of cases, while cervical cancer exhibits an even higher percentage of approximately 90%. The underlying mechanisms driving the differential occurrence of LOH in HLA expression between leukemic cells and solid tumors remain unclear. However, it is widely recognized that immune evasion represents a fundamental characteristic of cancer [19]. The aim of the majority of cancer immunotherapies is to counteract immune evasion by enabling T cells to target cancer cells. However, the majority of the population remains unbenefited from immunotherapies. This emphasizes the importance of identifying genomic and molecular determinants that stimulate immune evasion.

Impact of HLA loss on cancer therapy

Recent research highlights the significance of T-cell-mediated cytolytic activity against cancerous neoantigens. It is also important to consider the efficacy of immune checkpoint inhibition [20]. Antigenic presentation is reduced due to the downregulation of HLA genes, resulting in the facilitation of immune evasion. Immunohistochemistry or monoclonal antibodies have shown that HLA downregulation is prevalent in a range of cancers and is also linked with poor outcomes [21]. Trans et al. studied the LOH at HLA-C*08:02 in a tumor, which developed into a resistant lesion previously treated with tumor-infiltrating T lymphocytes. The HLA-C*08:02 allele is required for identification by the immune system and enables the presentation of the KRAS G12 neoantigen. The resected lesion lacks the HLA-C*08:02 molecule on chromosome 6, a major histocompatibility molecule. The loss of the HLA-C*08:02 molecule provides an immune escape mechanism for the tumor. However, the use of CD4+ cells targeting the mutant KRAS is an effective anti-tumor immunotherapy against cancer. The variation in HLA-A*0201 augments the risk of developing CLL. Moreover, the malfunctioning of T cells also enhances the progression of the disease [22]. The mechanism of immune evasion in progressive lesions is the deprivation of the HLA-C allele.

HLA LOH in non-small cell lung cancer (NSCLC)

**Figure 1:**
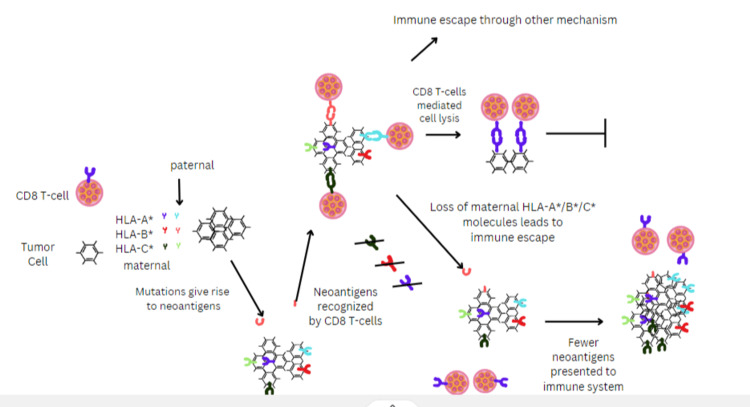
Non-small cell lung cancer (NSCLC) model for loss of human leukocyte antigen (HLA) allele. This model illustrates the relationship between the loss of heterozygosity of the HLA allele and immune escape in tumors.

The escape from immune predation in NSCLC is attributed to the loss of recognition of productive tumor neoantigens (Figure 1). An essential part of neoantigen presentation is the HLA class I molecule, which presents the antigen epitope to T cells. Moreover, HLA homozygosity is a mechanism behind immune escape. The process of tumor development involves the accretion of neoantigens, inducing local immune infiltrates of CD8+ T cells. These CD8+ cells act as a particular obstacle for tumors. Sub-clones with HLA LOH evade immune predation by escaping T-cell recognition [23].

Alteration in HLA phenotype in different malignancies

Multiple studies have been conducted to investigate abnormalities in HLA molecules, both classical and non-classical, in patients with CLL characterized by the accentuation of CD-5 cells, which are associated with malignant B-lymphocytes. While some patients with CLL exhibit no symptoms, others may require early therapeutic interventions [24]. Approximately 90% of CLL tumors exhibit HLA defects [25]. Various mechanisms contribute to the downregulation of HLA molecules, including genetic modifications. One prominent modification occurs on chromosome 6p21.3, which is located within the HLA locus and encompasses several genes. Among these genes are those encoding transporters involved in antigen processing. Extensive research has identified numerous chromosomal mutations responsible for the downregulation of HLA, such as alterations in genes that express proteins associated with latent membrane formation, as well as changes in the beta-2-microglobulin (β2m) locus on chromosome 15q21. Collectively, these findings underscore the significance of chromosomal alterations in suppressing HLA expression [26].

The genes present on chromosome 6p21 undergo downregulation and irreversible mutation resulting in inactivation, leading to loss of HLA. This process involves a two-step mechanism. Firstly, mutations occur in two genes responsible for encoding β2m, and secondly, the second copy is lost due to the deletion of genes in the 15q21 region. Additionally, the mutation and downregulation of the antigen-presenting machinery contribute to the regulation of HLA surface expression. The LOH is accounted for by the deletion of genes in the 16p21 region, specifically involving microsatellite markers [27].

Impact of LOH on immunotherapy

Rafael et al. studied progressive lesions in cancer patients who underwent immunotherapy to detect the LOH at 15q21 (also known as LOH-15q21) [28]. HLA class I expression in these tumors is characterized by a reduction in the β2-microglobulin (β2m) protein in the LOH15q21-positive tumors. These tumors carry only one intact β2m gene. Shrout et al. [29] demonstrated an intriguing finding in colorectal carcinoma. According to their study, colorectal carcinoma has decreased expression of β2m mRNA, associated with poor prognosis and metastasis. Additionally, the loss of HLA heterozygosity is also referred to as the modification of chromosome number 6 at 21 loci of the HLA (LOH-6p21). Maleno et al. [30] observed a high prevalence of LOH-6p21 in laryngeal cancer and a lower frequency in renal tumors. Therefore, the deprivation of genetic makeup in chromosomes 6p21 and 15q21 has affected the phenotype of HLA in tumor cells and their impact on immunogenicity.

Mechanism of immune evasion by HLA-G

There are multiple effects involved in the development of cancer, such as suppressing the dissolution of immune cells, prompting programmed immune cell death, generation of regulatory T cells (regT cells) through receptor binding, and impeding chemotaxis of immune effector cells [31]. Various receptors on HLA-G, including ILT2 (a cluster of differentiation 85j), ILT4/CD85d, CD8, and CD160, have been delineated. B cells express ILT-2. ILT-2 receptors are also present in NK cells, T lymphocytes, and dendritic cells. However, ILT-4 is present only in monocytes, neutrophils, and dendrites. The surface cytotoxic T cells express CD8+. Additionally, it is also present in NK cells [32]. Additionally, HLA-G augments the expression of matrix metalloproteinase, which is a tumor metastasis-related factor, providing greater effects on tumor progression [33]. HLA-G plays a crucial role in reconciling the inhibition of both innate and adaptive immune responses. This inhibition depends on the interaction of HLA-G with the receptors present in the different cell types. By such interactions, NK cells, T cells, B lymphocytes, polymorphonuclear leukocytes, and antigen-presenting cells are suppressed by HLA-G [34].

HLA-G-induced immunosuppression

The HLA-G-induced immediate immunosuppression includes the hindrance of CTL and NK cell lysis. Additionally, it hampers the multiplication of allogeneic CD4+ lymphocytes and triggers the apoptosis of CD8+ and NK cells as part of an immune evasion process, involving programmed cell death and suppression of cytotoxic T lymphocytes [35]. HLA-G interaction with the ILT2 inhibitory receptor inhibits interferon-γ production. Moreover, it affects the maturation, migration, and antigenic presentation of dendritic cells with T and NK cells [36,37]. In both dependent and independent B cell activation models, soluble HLA-G inhibits B-lymphocyte multiplication and suppresses immunoglobulin IG by interacting with the ILT2 receptor [38]. The mechanism of cytolysis employed by neutrophils, involving phagocytosis and the generation of reactive oxygen species, is impeded by the interaction of HLA-G5 with the ILT4 receptor [39].

Impact of HLA on targeted therapies in CLL

The significant immune suppression in CLL increases the susceptibility to infections, autoimmune diseases, and secondary malignancies. The immunosuppressive nature of CLL involves intricate mechanisms that allow tumor evasion. These mechanisms encompass the expression of CD2000, HLA-G, and programmed death ligand (PD-L1) [40,41]. Additionally, B cells in CLL contribute to immunosuppression by producing IL-10, a cytokine known for its inhibitory effects on the immune system [42]. Dysfunctionality of T cells further exacerbates the immune challenges in CLL. Current treatments that aim to enhance cell-mediated immunity in CLL are limited. However, Burton's tyrosine kinase (BTK), such as ibrutinib has shown promising response rates and substantial remission in patients with CLL [43].

Effect of BTKi ibrutinib on HLA-DR and T cells

The study conducted by Manukyan et al. investigated the long- and short-term effects of ibrutinib on the expression of HLA-DR in various cell types, including CLL cells, T lymphocytes, and other white blood cells. The research included a sample of sixteen individuals previously diagnosed with CLL who received treatment with ibrutinib. Following treatment with ibrutinib, there was a noticeable decrease in the population of cells expressing HLA-DR, alongside an increase in the quantity of CLL cells. Additionally, both the protein and messenger RNA levels of HLA-DR exhibited a decrease in expression when the culture of CLL patient cells was exposed to ibrutinib. However, one month after the treatment, there was an increase in the population of CD4+ and CD8+ T cells, along with elevated HLA-DR expression in both CD4+ and CD8+ cells. Moreover, an augmentation in the population of CD4+ cells with HLA-DR expression and an elevation of HLA-DR expression in monocytes were observed [44]. In both in vivo and in vitro research, it was found that the induction of T cell kinase by ibrutinib, an irreversible inhibitor of the interleukin-2 receptor, resulted in an escalated Th1 reaction. This increase in the population of CD4+ and CD8+ T cells occurred through a BTK-independent mechanism [45]. The immune cells, including T lymphocytes, CD4+, and CD3+ cells, notably increase in the blood of relapse or refractory and unaffected chronic lymphoma leukemia patients, leading to a decrease in the CD4:CD8 ratio [46-48]. Additionally, ibrutinib, by hindering cell kinase activity through interaction with the ILT-2 receptor, can diminish activation-induced cell death. It further results in a temporary augmentation in circulating T-cell counts [49].

HLA-G-based targeted therapy

**Figure 2:**
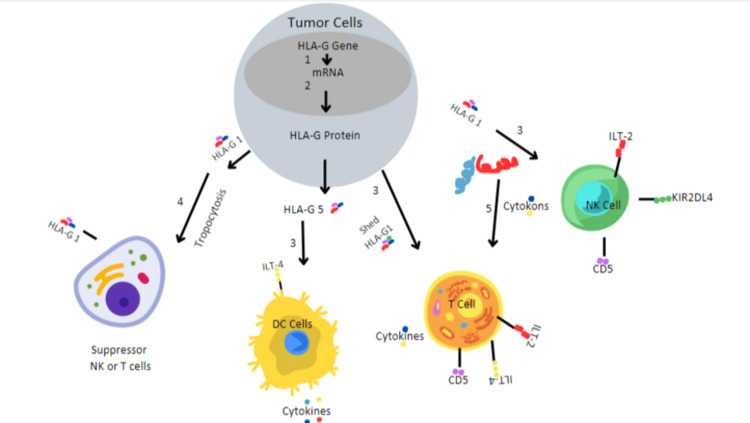
HLA-G as immunotherapy targets for anticancer treatment. (1) Stop HLA-G transcription. (2) HLA-G mRNA translation. (3) Inhibition of the link between HLA-G on tumor cells and inhibitory receptors on T and NK cells. (4) Trogocytosis. (5) Stimulation of antigen-specific immune response by HLA-G idiotype antibodies. HLA: human leukocyte antigen, NK: natural killer.

Patients with B cell lymphocytic leukemia (B-CLL) who exhibit higher levels of HLA-G are associated with shorter survival periods. Conversely, those with fewer than twenty-three percent HLA-G positive B cells tend to experience longer survival rates [50]. Moreover, HLA-G is a better diagnostic factor than ZAP-70, as the use of HLA-G is the primary factor for predicting disease progression, and serum beta-2-microglobulin and associated protein-70 (ZAP-70) are secondary factors [51]. However, HLA-G is responsible for the immune escape mechanism; therefore, several novel therapies are targeted at the HLA-G molecule. Potential therapies that reduce the presence of HLA-G in tumors or suppress its transcription prove to enhance the efficacy of NK cells in lysing tumor cells, especially in patients with CLL. Antibodies, particularly targeting HLA-G1, obstruct the ability of NK cells to eliminate tumor cells, highlighting HLA-G's role in immune evasion. By bolstering CLL tumor cells against NK cell-induced killing, HLA-G actively contributes to the immune escape of tumor cells in vivo. Consequently, it is inferred that HLA-G serves as a protective mechanism shielding CLL cells from cleavage by NK cells [52]. HLA-G obstructs the interconnection between HLA-G and immune cell HLA-G receptor- Ig-like receptor. To reinstate the proliferation of T cells, antibodies are directed against ILT-2 and ILT-4 [53]. The stimulation of the immune system is possible by utilizing an immunogenic peptide derived from HLA-G. HLA-G146-154 is an HLA-G-derived peptide that activates peptide Cytotoxic T lymphocytes, which can serve as a potential agent of anti-cancer immunotherapy [54]. Antibodies neutralizing the HLA-G molecule, and soluble recombinant molecules to neutralize the suppressive effects of HLA-G in immune cells are being explored. The recombinant molecules mostly used are Leukocyte-Ig-like receptor 1, leukocyte-Ig-like receptor-2, and Fas. Antibodies against HLA-G molecules present on the exterior of malignant cells, in fusion with other anti-cancer drugs, are essential for anti-tumor activity. These agents explicitly target cancer cells, escalating therapeutic effects and curtailing the adverse effects of the drug (Figure 2).

## Conclusions

Based on the findings, it is concluded that the LOH of the HLA allele enables tumor cells to evade dissolution mediated by natural killer cells. The clinical importance of HLA-G as a potential agent in cancer drug discovery cannot be neglected. However, some studies contradict the notion that T-cell immune evasion is due to LOH in the HLA molecule. If LOH is not the culprit for immune evasion, then the tumor microenvironment should be further studied. The prediction of ICB treatment effectiveness is an important marker for LOH. The relapse in chronic lymphoma leukemia patients is attributed to HLA antigen changes. The expression of the HLA class I molecule is a precondition for successful immunotherapy for leukemia. HLA-G is involved in tumor evasion by hindering cytolysis, suppressing the stimulation of cytokines, inducing apoptosis of immune cells, and impairing chemotaxis. The resistance to immunotherapies is due to the suppression of immune checkpoints, LOH in HLA, and interferon-related mechanisms.
